# Bayesian mediation modeling of racial disparity for maternal birth outcomes in United States

**DOI:** 10.18103/mra.v12i9.5858

**Published:** 2024-09

**Authors:** James Thompson

**Affiliations:** College of Veterinary Medicine and Biomedical Science, Texas A&M University, College Station TX USA 77843-4475

**Keywords:** maternal, health disparity, mediation, Bayesian, body mass index, gestational weight gain

## Abstract

**Background:**

In the United States, racial disparities for adverse maternal health outcomes persist, and the causes remain unknown. The disparities for women of Black ethnicity include increased risk of gestational hypertension, hypertension eclampsia, cesarean section, and admission to an Intensive Care Unit, and reduced risk of parturition induction. Without evaluating racial disparity, studies identify one cause of these conditions as the interaction between pre-gestational body mass index and gestational weight gain. What has not been determined is how body mass index and gestational weight gain contribute to racial disparity. The study's objective was to determine if the interaction between body mass index and gestational weight gain can explain the racial disparity in five maternal outcomes.

**Methods:**

The approach involved mediation analysis by performing Bayesian estimation of potential outcomes for each combination of causes. Causes included risk of Black ethnicity, body mass index, and gestational weight gain.

**Results:**

Improving both body mass index and gestational weight gain to what is considered optimal would increase the racial disparity for gestational hypertension by 19.2%, have a non-significant effect on racial disparity for hypertension eclampsia, reduce the racial advantage for Black women receiving induction by 16.9%, and reduce the racial disadvantage for delivery by cesarean and admission to an Intensive Care Unit by 49.9% and 36.9%, respectively.

**Conclusion:**

Preventive programs can have a wide range of effects on racial disparity, from decreasing to increasing the disparity. Implementing the mediation evaluation approach illustrated here would optimize clinical decisions, guide public health policy, and eventually mitigate racial mistrust.

## Introduction

In the United States, racial disparities for adverse maternal health outcomes persist, with Black women experiencing a 3–4 times higher risk of maternal mortality and a 2–3 times higher risk of severe maternal morbidity. However, the causes of the disparities remain unknown.^1,2^ The disparities for women of Black ethnicity in the United States include increased risk of gestational hypertension, hypertension eclampsia, cesarean section, and admission to an Intensive Care Unit (ICU), and in the opposite direction, reduced risk of parturition induction.^3^ Without evaluating racial disparity, studies have determined one cause of these conditions is an interaction between pre-gestational body mass index (BMI) and gestational weight gain (GWG).^4^ What hasnť been determined is whether BMI and GWG cause the racial disparity. These factors could cause the disparity if either is more common among Black women or if the factor conveys more risk to Black women. Even though race cannot be altered, determining whether these interactions cause racial disparity can be resolved as a “mediation” question.^5^

The Institute of Medicine (IOM) guidelines recognize the interaction between BMI and GWG and recommend that normal weight women (BMI 18.5–24.9) gain 11.5–16 kg during pregnancy. Higher BMI women should gain less weight and lower BMI women gain more weight.^6^ In spite of the literature cited by the IOM to support a proactive response to the interaction, many studies evaluating obesity as a cause of adverse maternal outcomes use either GWG or BMI,^7^ with BMI estimated for either prepregnancy weight^8^ or weight at delivery.^9^ Assuming that a woman's height does not change during pregnancy, then any two of the three estimates (prepregnancy BMI, GWG, or delivery BMI) will provide the same information.

In mediation modeling, it has become common practice to include interaction between the cause of interest and a potential mediator.^5^ However, when multiple mediators are being evaluated, it has been impossible to resolve the complete set of direct and indirect effects.^5^ Until recently, attempts to model multiple mediators has depended on complex pathways defined by directed acyclic graphs (DAG), and the approaches have been too complex to resolve without extraordinary assumptions.^5,10–15^ The failure to evaluate interactions among causes and race might explain the lack of progress in identifying causes of the disparities for Black women. Two recent advances provide an opportunity to remedy this. The first innovation was a proposal to represent the interaction in a DAG as a unique node.^16^ Under this proposal, the interaction becomes a multivariate node, with each classification level of the node representing a unique combination of risks and the risk probability becoming the potential outcome. The second innovation was that when modeling under the assumptions of this DAG, estimating the potential outcome probabilities is simple using a Bayesian modeling approach.^17^

The study objective was to determine if BMI and GWG can explain the maternal racial disparity in five outcomes. To that end, the study evaluates how these two potential mediators interact and implements Bayesian estimation of potential outcomes for each combination of causes, with the causes including risk of race for women of Black ethnicity and the two potential mediators.

## Methods

### DATA

The source of data was the National Vital Statistics System which provides data online with all patient identifiers removed (https://www.cdc.gov/nchs/nvss/births.htm). In the United States, state laws require birth certificates for all births, and federal law mandates national collection and publication of births and other vital statistics data. The National Vital Statistics System, the federal compilation of these data, is the result of the cooperation between the National Center for Health Statistics (NCHS) and the states to provide access to statistical information from birth certificates. The website also describes standard forms for the collection of the data and model procedures for the uniform registration of the events. The retrieved data for the current study included all singleton births from 2016 to 2021.

### MEDIATION VARIABLES

Race was the cause of interest (X) and classified as “Black” (X=2) for all women of Black ethnicity and “Non-Black” (X=1) for all other ethnicities and races. The potential mediators included prepartum BMI and adjusted GWG, whereby a linear correction factor was used to correct GWG to 40 weeks of gestation. Deleted records included patient records missing one or more outcomes or missing data required for estimating BMI and GWG. Prepartum BMI was coded M1 = 1 (for “optimal”) for BMI of 18.5 to 25 and M2=2 otherwise (for “non-optimal”). Adjusted GWG was coded M2=1 (for “optimal”) for a gain of 11.5 to 16 kg and M2=2 otherwise (for “non-optimal”). Five outcomes, referred to as maternal outcomes were modeled, including gestational hypertension, hypertension eclampsia, parturition induction, delivery by cesarean section, and admission to an ICU. Outcomes (Y) were Y= 0 for non-occurrence and Y=1 for occurrence for each of the maternal outcomes.

### MODEL

**Figure 1.**
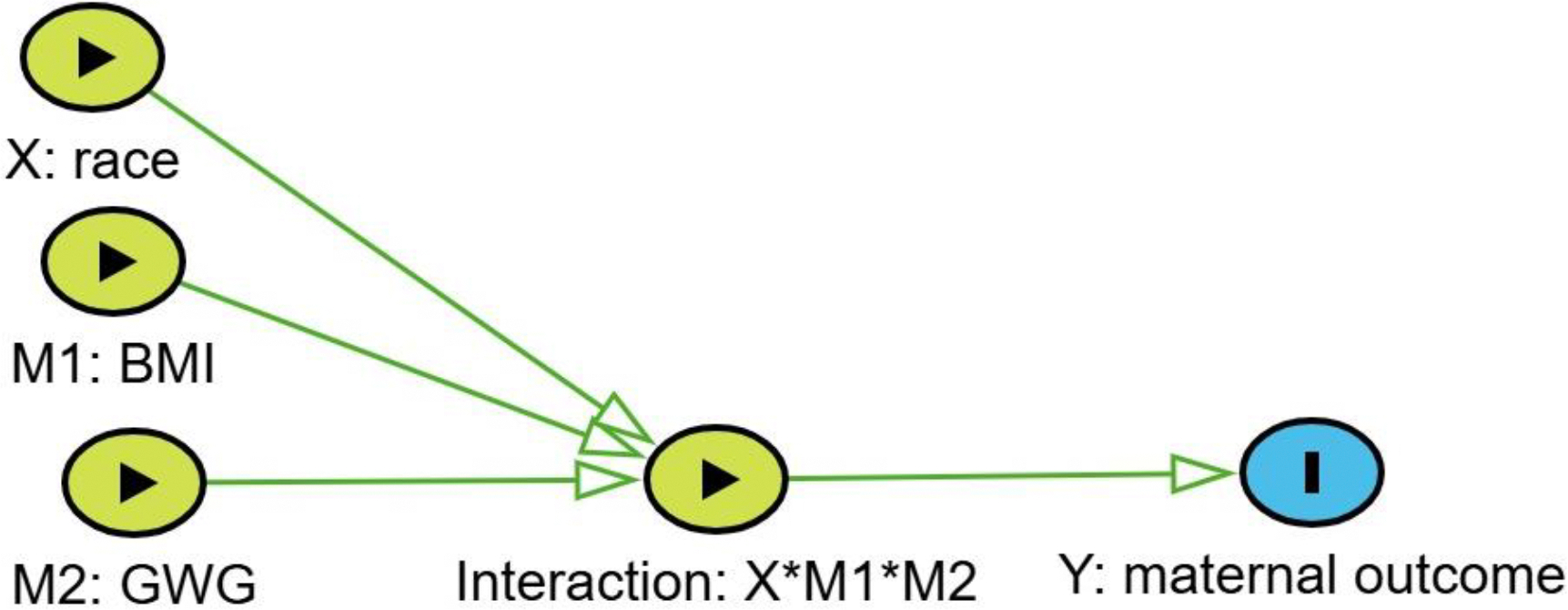
Directed Acyclic Graph. Each of the three binary nodes has a single arrow to the interaction node, and the interaction node has eight classification levels.

A DAG that contained a node representing interactions among a cause (X) and two mediators (M1 and M2) guided the modeling. The interaction node was an eight-level multivariate node representing the disease rates for all combinations of the cause and mediators (Figure 1).

### BAYESIAN ESTIMATION OF POTENTIAL OUTCOMES

The model estimated rates for potential outcomes using direct Bayesian estimation. The data contained i = 8 rows of data, with each row identified by unique values for X and two mediators (M1 and M2). Each row contained a count of incident cases $\left(r_{i}\right)$ and births $\left(n_{i}\right)$. The modeling fit the count of cases $r_{i}$ as binomial with a rate parameter $PO\left[X_{i},M1_{i},M2_{i}\right]$ and the count of births $n_{i}$:

$$r_{i}∼Binomial\left(PO\left[X_{i},M1_{i},M2_{i}\right],n_{i}\right)\text{.}$$

The rate parameters were the potential outcomes, given Umform(0,1) priors:

PO[1:2,1:2,1:2]~Uniform(0,1).

Estimation of the total effect (TE) used the standard counterfactual definition:

Prob(Y)|X=2−Prob(Y)|X=1.

Estimation of controlled direct effects (CDE) used standard counterfactual definitions for individual mediators and all combinations of mediators (Table 1).

The percent attributable (PA) was the difference between TE and CDE expressed as a percent of TE:

PA=100*(TE−CDE)/TE.

Specifying minimally informative prior values avoided influencing the posterior distributions. The prior for each potential outcome was an equal probability for the entire range of 0 to 1. Estimation was performed using MultiBUGS.^18^ A burn-in of 5000 iterations was discarded, and the next 10,000 iterations were collected for posterior distributions. Convergence was determined by monitoring chains with disparate starting values. Reported results are the median and 95% credibility intervals which were the 2.5 and 97.5 percentiles taken directly from the posterior distributions. A result was defined as non-significant if the 95% credibility interval included zero. The code used for MultiBUGS and data are available from the author upon request.

## Results

The downloaded data contained 18,364,229 records of singleton births. None of the records was missing the mother's ethnicity. Among the deleted records, 44,081 (0.2%) were deleted because the record was missing one or more of the five outcomes, and 614,965 (3.3%) were deleted because they were missing one or more variables needed to calculate GWG and BMI (height, weight at time of birth, prepregnancy weight, obstetric estimate for weeks of gestation). This left 17,705,183 observations for modeling.

The combinations of BMI and GWG showed different prevalence probabilities for women of Black ethnicity than the rest of the population (Table 2). Black mothers were more likely to have non-optimal BMI and GWG (51.3% versus 41.7%) and less likely to have optimal BMI and GWG (9.7% versus 15.1 %). It was more common for Black women to have optimal GWG with non-optimal BMI (14.7% versus 14.0%) and less common for Black women to have optimal BMI with non-optimal GWG (24.2% versus 29.2%).

The racial disparities for five outcomes are shown in Table 3. In the table, a negative under racial disparity for parturition induction means Black women were less likely to have induction. A negative value under percentage eliminated means the risk for Black women increased. Four maternal outcomes (gestational hypertension, hypertension eclampsia, delivery by cesarean section, and admission to ICU) were more common for women of Black ethnicity, while parturition induction was less common. Table 3 also shows the percentages of racial disparity attributable to BMI and GWG.

Optimizing both BMI and GWG would increase the racial disparity for gestational hypertension by 19.2%. The percentage of disparity eliminated was a negative value of −19.2% (−28.6,−10.2). The change in racial disparity for hypertension eclampsia was non-significant. The racial disparity for induction would become less favorable for Black women by 16.9% (10.2, 23.6). Delivery by cesarean and admission to ICU would reduce racial disparity by 49.9% (45.3, 54.6) and 36.9% (12.7, 59.2), respectively. For the preventive scenario of optimizing BMI and not addressing GWG, the racial disparity would increase for gestational hypertension, with a negative percent eliminated of −8.7% (−15.1, −2.5). The change would result in a non-significant change for hypertension eclampsia and parturition induction. Racial disparity would decrease for cesarean delivery and admission to ICU by 57.6% (55.1, 60.2) and 23.8% (8.0, 38.0), respectively. Optimizing GWG would increase racial disparity for gestational hypertension and cesarean delivery by −34.3% (−42.6, −26.4) and −14.2% (−17.9, −10.7), respectively. The change would have a non-significant effect on hypertension eclampsia and admission to ICU. Parturition induction would decrease in the extent it favors Black women by 21.0% (16.4, 25.5).

## Discussion

### CHALLENGE

The continuing challenge is to identify the causes of racial disparity in the United States for pregnancy outcomes. The current study suggests two important explanations for the ongoing racial disparity.

First, the study shows that mediation modeling should be able to predict that preventive programs can affect racial disparity over a broad range, from increasing to decreasing disparity. For example, improving both BMI and GWG to what is defined as optimal would increase the racial disparity for gestational hypertension by 19.2%, have a non-significant effect on racial disparity for hypertension eclampsia, reduce the racial advantage for Black women receiving induction by 16.9%, and reduce the racial disadvantage for delivery by cesarean and admission to an ICU by 49.9% and 36.9%, respectively. Clearly, it is possible that preventive programs have been less effective for Black women than women of other races and ethnicity. Knowledge of this potential is essential for “cultural awareness.” Furthermore, knowledge of race-specific risks for potential mediators is fundamental to clinical competence. Most importantly, failure to consider this potential when planning public health responses could constitute structural racism.

Second, the study shows that interaction among multiple mediators is important. For example, optimizing BMI would reduce racial disparity for cesarean section by 40.2%, but optimizing GWG would increase that racial disparity by 14.2%, and the combination of optimizing both would decrease racial disparity by 49.9%. The results reveal multiple examples of interaction between BMI and GWG. This should not be surprising, because the study evaluated two factors with a high prior likelihood of interaction. However, the likelihood of an interaction is not necessary for justifying the approach. Long ago, Pearl recommended that estimating the effect of a cause can be completed only at a specific value of the mediator.^19^ This clearly extends to each observed combination of multiple classification mediators. In mediation modeling, the interaction among multiple mediators must be included in the analysis without statistically testing for its inclusion.

### OPPORTUNITY

The National Institute on Minority Health and Health Disparities (NIMHD) supported a special issue that focused on the causes of racial health disparities in the United States.^20,21^ The authors concluded that novel methods are necessary to identify causes whose manipulations could form the bases of preventive care programs.^22,23^ The causes of racial disparity are largely unknown but virtually certain to be multifactorial. What was needed was a mediation model that could model interactions among multiple mediating causes. This is now possible using Bayesian estimation of potential outcomes, as illustrated here.

Studying the causal effects of multiple mediators upon multiple outcomes can lead to confusing results. More research should address this limitation by focusing on the clinically most relevant outcome, in this case, maternal mortality.^24^ The five morbidities modeled in the current study could be assumed to interact as causes of maternal mortality. Evaluating the mediation of maternal mortality by maternal morbidities and other causes will glean considerable information on the causes of the morbidities. The assumption that the mediators interact through a common multivariate interaction node, combined with the simplicity of estimating each potential outcome, provides an exciting opportunity for Bayesian modelers.

The current study evaluated a single race group against the rest of the population. Evaluating racial disparities among all race groups would be more appropriate. These disparities can be modeled by comparing each individual race to the overall average risk, thereby permitting evaluation of the disparities affecting a single race.^3^ With this “one at a time” approach, it will not be necessary to model how a six-category race variable interacts with complex sets of mediating causes.

The current study grouped multiple levels of BMI and GWG into the single classification of “non-optimal.” This was a convenience for the purpose of promoting discussion. The clinically optimal approach would model the potential outcomes using a wide range of categories for both BMI and GWG. Estimation of these potential outcomes must be race specific. Such estimates would allow individual patients to consider what changes are possible for their BMI and GWG and how these changes would affect their risk, thereby increasing patient engagement. Most of all, it would improve patient-provider relationships by increasing both cultural and clinical competency.^1^

Besides different rates between the two race groups for both prevalence of mediators and race-specific risks, there is a difference in the acceptance of public health programs. A legacy of racial discrimination in medical research and the health care system has been linked to a low level of trust in medical research and medical care among African Americans.^25^ This mistrust is associated with perceived discrimination, and addressing this perception is one of the key elements of addressing racial health disparities.^26^

## Conclusion

Preventive programs can have a wide range of effects on racial disparity, from decreasing to increasing the disparity. Implementing the mediation evaluation approach illustrated here would optimize clinical decisions, guide public health policy, and eventually mitigate racial mistrust.

## Figures and Tables

**Table 1.** Definitions of controlled direct effect (CDE). The counterfactual definition of CDE defines the probability of infant mortality P(Y) conditional upon specific values for race (X) and mediators M1 (prepartum BMI), and M2 (GWG). The model estimator is the value from the model that estimates the CDE.

Mediator	Counterfactual Definition of CDE	Model Estimator of CDE ^1^

M1	P(Y)|X=2, M1=1-P(Y)|X=1, M1=1	PO [2,1,NA]-PO [1,1,NA]
M2	P(Y)|X=2, M2=1-P(Y)|X=1, M2=1	PO [2,NA,1]-PO [1 ,NA,1]
M1 ,M2	P(Y)|X=2, M1=1, M2=1-P(Y)|X=1, M1=1, M2=1	PO [2,1,1]-PO [1,1,1]

1 PO[a,b,c] is the potential outcome for: a (race) equals 1 for Non-Black and 2 for Black; b (prepartum BMI) equals 1 for ideal and 2 for non-ideal; c (GWG) equals 1 for ideal and 2 for non-ideal. A value of NA means the indicator value is treated as missing.

**Table 2.** The percent probability of observing combinations of optimal prepartum BMI and optimal GWG.

Mediators		Cause (Ethnicity)	
BMI	GWG	Non-Black	Black

Optimal	Optimal	15.1 (15.1, 15.1)	9.7 (9.7, 9.8)
Non-optimal	Optimal	14.0 (14.0, 14.0)	14.7 (14.7, 14.8)
Optimal	Non-optimal	29.2 (29.2, 29.3)	24.2 (24.2, 24.3)
Non-optimal	Non-optimal	41.7 (41.6, 41.7)	51.3 (51.3, 51.4)

**Table 3.** Racial disparity among five outcomes. The total effect is multiplied by 1000 and represents the difference in number of cases comparing 1000 women in each race group.

Maternal Outcome	Racial Disparity	Percentage of Racial Disparity Eliminated if BMI and/or GWG Optimized		

	Total Effect (× 10^3^)	Optimize all BMI	Optimize all GWG	Optimize all GWG and BMI

Gestational hypertension	10.5 (10.1 –10.8)	−8.7 (−15.1, −2.5)	−34.3 (−42.6, −26.4)	−19.2 (−28.6, −10.2)
Hypertension eclampsia	1.1 (1.0, 1.2)	−1.4 (−15, 10.5)	−10.0 (−26.1, 4.9)	9.6 (−9.6, 27.3)
Parturition induction	−28.1 (−28.7, −27.5)	0.1 (−4.1, 4.1)	21.0 (16.4, 25.5)	16.9 (10.2, 23.6)
Delivery by cesarean	40.2 (39.6, 40.9)	57.6 (55.1, 60.2)	−14.2 (−17.9, −10.7)	49.9 (45.3, 54.6)
Admission to ICU	0.7 (0.7, 0.8)	23.8 (8.0, 38.0)	9.9 (−8.8, 26.9)	36.9 (12.7, 59.2)
